# Rheological and Structural Characterization of Carrageenans during Depolymerization Conducted by a Marine Bacterium *Shewanella sp*. LE8

**DOI:** 10.3390/gels10080502

**Published:** 2024-07-28

**Authors:** Xiong Li, Chuyi Li, Yizhou Liu, Gang Han, Congyu Lin, Xiaoli Chen, Jian Mao

**Affiliations:** 1 Guangdong Engineering Research Center of High-Value Utilization and Equipment Development of Marine Biological Resources, Southern Marine Science and Engineering Guangdong Laboratory (Guangzhou), Guangzhou 511458, China; boyyerli@163.com (X.L.); cxlgdut@163.com (X.C.); 2 College of Life Science, Fujian Agriculture and Forestry University, Fuzhou 350002, China; chuylee@163.com; 3 School of Environmental Science and Engineering, Tianjin University, Tianjin 300350, China; liuyizhou@tju.edu.cn; 4 School of Food Science and Technology, Jiangnan University, Wuxi 214122, China; 6220112135@stu.jiangnan.edu.cn (G.H.); lincongyu@jiangnan.edu.cn (C.L.)

**Keywords:** rheological property, carrageenan, structural characterization, *Shewanella*, depolymerization

## Abstract

Carrageenans were widely utilized as thickening and gelling agents in the food and cosmetic industries, and their oligosaccharides have been proven to possess enhanced physicochemical and biological properties. In this study, *Shewanella sp.* LE8 was utilized for the depolymerization of κ-, ι-, and λ-carrageenan under conditions of fermentation. During a 24-h fermentation at 28 °C, the apparent viscosity of κ-, ι-, and λ-carrageenan decreased by 53.12%, 84.10%, and 59.33%, respectively, accompanied by a decrease in storage modulus, and loss modulus. After a 72-h fermentation, the analysis of methylene blue and molecular weight distribution revealed that ι-carrageenan was extensively depolymerized into smaller polysaccharides by *Shewanella sp.* LE8, while exhibiting partial degradation on κ- and λ-carrageenan. However, the impact of *Shewanella sp.* LE8 on total sugars was found to be limited; nevertheless, a significant increase in reduced sugar content was observed. The ESIMS analysis results revealed that the purified components obtained through ι-carrageenan fermentation for 72 h were identified as tetrasaccharides, while the two purified components derived from λ-carrageenan fermentation consisted of a hexasaccharide and a tetrasaccharide, respectively. Overall, the present study first reported the depolymerization of ι-and λ-carrageenan by *Shewanella* and suggested that the *Shewanella* could be used to depolymerize multiple carrageenans, as well as complex polysaccharides derived from red algae, to further obtain their oligosaccharides.

## 1. Introduction

Carrageenan is a linear sulfate-substituted polysaccharide extracted from the cell wall of carrageenophyte marine red algae in an environmentally friendly way, characterized by the alternately linked D-galactose with α-1,3 and β-1,4 linkages as its fundamental structure [1]. The classification of carrageenan encompasses over 10 types, which are determined by the number and position of sulfate substitutions and with or without anhydrogalactose. Among these types, κ-, ι-, and λ-carrageenan are the most commercially exploited. The excellent properties of gel-forming ability and chemical stability have made it widely utilized as a thickening and gelling agent in the food and cosmetic industries [2]. Moreover, they have been demonstrated to possess a wide range of pharmaceutical properties, encompassing immunomodulatory, antitumor, anticoagulant activities, anti-hyperlipidemic effects, and in vitro antiviral effects [3]. However, the high molecular weight (MW), intricate structure, and potentially adverse effects associated with these compounds have imposed limitations on their development and applications [4].

Derivatization is a crucial method for altering the physical and chemical properties of carrageenan, thereby enhancing its solubility and bioavailability. The depolymerization of carrageenans can be achieved through various preparation methods, including physical degradation techniques such as radiation or microwave assistance [5,6], chemical degradation methods like acid hydrolysis [7], and enzymatic hydrolysis [8,9,10]. In addition, carrageenan or its derived oligosaccharides can also undergo chemical modifications such as oversulfation, desulfation, acetylation, or phosphorylation to enhance their physicochemical and biological properties [11,12]. Microbial degradation can be considered as a component of enzymatic degradation due to the shared mechanism of degradation. These processes offer advantages such as operational simplicity, cost-effectiveness, and efficiency, making them potential methods for carrageenan derivatization. Furthermore, they are particularly advantageous for complexes with multiple structures. Since the currently reported enzymes are specific for only one type of carrageenan and can be classified into κ-carrageenase (EC 3.2.1.83), ι-carrageenase (EC 3.2.157), and λ-carrageenase (EC 3.2.1.-) [13]. The degradation of carrageenan by microorganisms has been extensively documented in numerous scientific studies, leading to the development of correspondent enzymes. The κ-carrageenase-related microorganisms are the most widely distributed, including some of the species in the genera *Pseudoalteromonas*, *Pseudomona*, *Tamlana*, *Vibrio*, *Pedobacter*, *Zobellia*, *Cellulophaga*, *Cytophaga*, etc., while the ι-carrageenases-related microorganisms belong to several marine bacteria, such as *Alteromonas*, *Cellulophaga*, *Microbulbifer*, *Flavobacterium*, and *Wenyingzhuangia* [13]. Only a few λ-carrageenase -related microorganisms have been reported, including some species in the genera *Pseudoalteromonas*, *Bacillus*, *Maribacter*, and *Wenyingzhuangia* [14,15,16,17,18]. The genus *Shewanella* encompasses a diverse group of facultative anaerobic bacteria that are widely distributed in marine and freshwater environments [19]. Furthermore, only a limited number of strains have been documented to possess the capability of enzymatically degrading κ-carrageenan [20,21]. Despite the availability of genome sequences and submissions to the NCBI database in previous reports, detailed investigations on the versatile polysaccharide degradability of *Shewanella* species remain scarce [22].

Carrageenan oligosaccharides are the most prevalent and extensively investigated polysaccharides derived from carrageenan. Numerous studies have demonstrated that carrageenan oligosaccharides exhibit a wide range of biological activities beneficial to human health, including antioxidant, antitumor, immunomodulatory, anti-inflammatory, and antiviral properties, and have obtained increasing attention in various potential application fields [23]. Despite the extent of degradation that may have negative effects on the gelling properties of degraded carrageenan itself, its enhanced activity presents potential for its application in gels. The studies have confirmed that the antitumor activity of carrageenan exhibits an inverse correlation with its molecular weight, wherein high-sulfonated oligosaccharides demonstrate superior antitumor properties compared to light-sulfonated or non-sulfonated samples [24]. Moreover, hydrogels with carrageenan oligosaccharides and antimicrobial agents have been proven to have antibacterial and anti-inflammatory properties for the treatment of periodontitis [25].

Therefore, in this study, we isolated a novel *Shewanella* strain from fermenting three representative carrageenans, namely κ-carrageenan, ι-carrageenan, and λ-carrageenan, under conditions of limited nutrients. The rheological properties were investigated to evaluate the potential degradation of carrageenans. Changes in molecular weight, total sugars, and reducing sugars during degradation were examined to assess their applicability for oligosaccharide preparation. Furthermore, the structure of carrageenan oligosaccharides was elucidated using ESIMS analysis. These findings will provide a fundamental basis for the production of depolymerized carrageenan and red algae polysaccharides, as well as their oligosaccharides by *Shewanella*.

## 2. Results and Discussion

### 2.1. The Apparent Viscosity of Carrageenans after LE8 Fermentation

**Figure 1:**
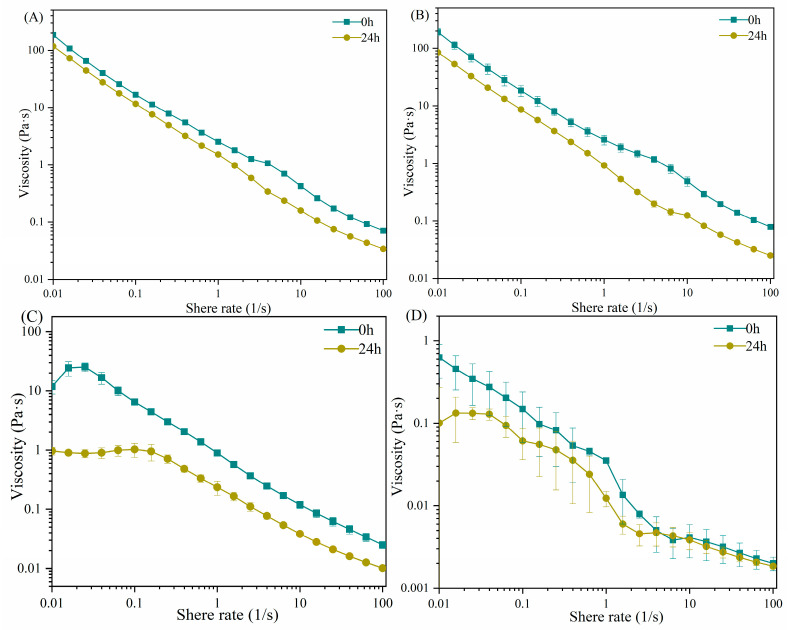
The apparent viscosity of fermentation medium: (**A**) carrageenan (C107615), (**B**) κ-carrageenan, (**C**) ι-carrageenan, (**D**) λ-carrageenan.

The variation in apparent viscosity is indicative of changes in intermolecular forces, whereby a higher apparent viscosity corresponds to stronger molecular attractive forces [26]. The flow curves of apparent viscosity as a function of shear rate are illustrated in Figure 1. The observed samples all demonstrated typical shear thinning behavior, wherein the apparent viscosity gradually decreased as the shear rate increased from 0.1 to 100 s^−1^. This phenomenon can be attributed to a decrease in physical interactions between adjacent polymer chains or structural breakdown [27]. The κ-carrageenan exhibited a higher apparent viscosity at the same shear rate, followed by ι-carrageenan, with λ-carrageenan displaying the lowest apparent viscosity. The observed phenomenon aligned with their ability to form gels when exposed to salt concentrations (about 2.5% NaCl) in the culture medium [28,29]. Furthermore, the carrageenan (C107615) demonstrated a comparable apparent viscosity to κ-carrageenan, suggesting that carrageenan (C107615) primarily consisted of κ-carrageenan. However, after 24 h of fermentation with LE8, the apparent viscosity of all carrageenan groups was decreased. Specifically, the apparent viscosity of ι-carrageenan exhibited a significant decrease of 84.10% (from 6.48 to 1.03 Pa·s at 0.1 s^−1^, *p* < 0.01), followed by λ-carrageenan with a reduction of 59.33% (from 0.15 to 0.061 Pa·s at 0.1 s^−1^, *p* < 0.05) and κ-carrageenan with a reduction of 53.12% (from 18.43 to 8.64 Pa·s at 0.1 s^−1^, *p* < 0.01). Additionally, the carrageenan (C107615) experienced a notable decrease in apparent viscosity by approximately 30.69% (from 16.78 to 11.63 Pa·s at 0.1 s^−1^, *p* < 0.01). The results revealed that enzymatic hydrolysis of certain glucoside bonds in carrageenan occurred during LE8 fermentation, leading to a reduction in the gelling properties and a significant decrease in viscosity.

### 2.2. Rheological Characterization of Carrageenans after LE8 Fermentation

**Figure 2:**
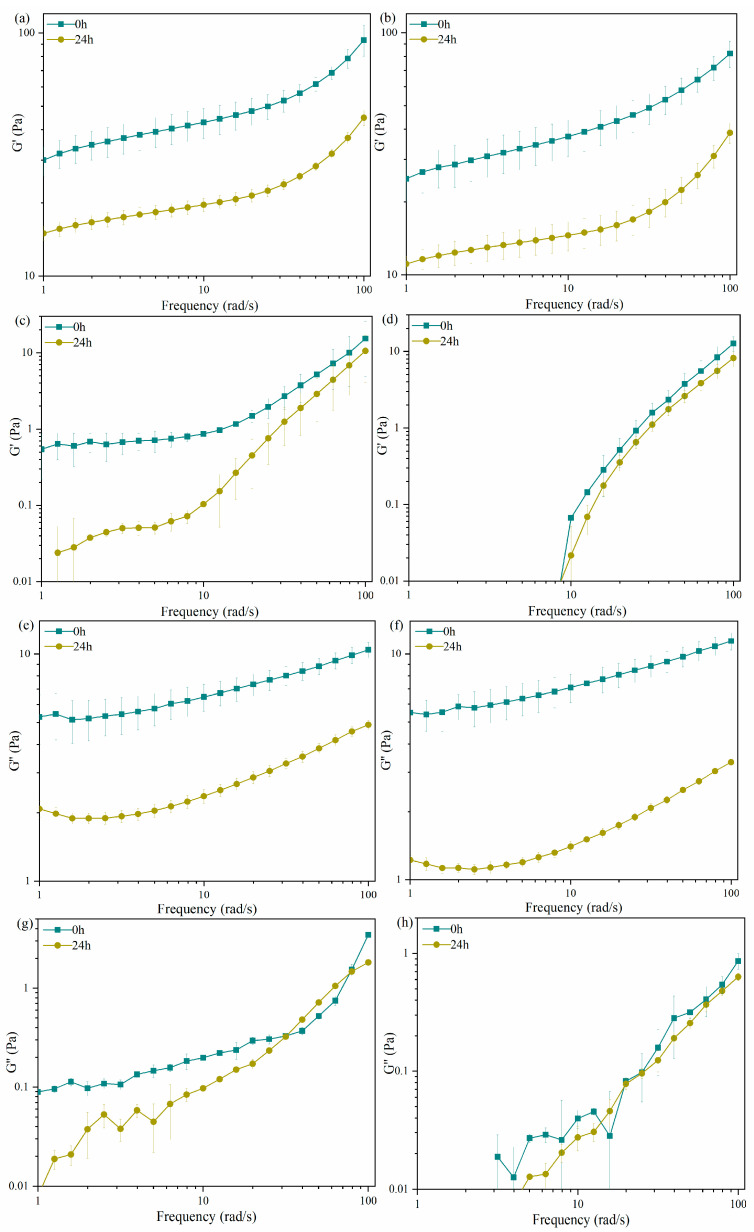
Rheological properties of fermentation medium: (**a**–**d**) storage modulus of carrageenan (C107615), κ-carrageenan, ι-carrageenan, and λ-carrageenan, respectively; (**e**–**h**) loss modulus of carrageenan (C107615), κ-carrageenan, ι-carrageenan, and λ-carrageenan, respectively.

Carrageenan is an anionic polysaccharide and is used in the food industry due to its thickening and gelling properties [30]. Both κ- and ι-carrageenans can form gels in the presence of monovalent and divalent ions, while λ-carrageenan yields only viscous solutions. However, λ-carrageenan is capable of forming gels with certain trivalent ions such as Fe^3+^ [31]. The frequency dependence of the moduli of carrageenan culture mediums before and after fermentation by LE8 is illustrated in Figure 2. The mechanical spectra of all the samples displayed typical gel-like behavior, with G′ > G″ across the entire frequency range examined. The culture media containing 0.2% κ-carrageenan or carrageenan (C107615) and 2.5% sea salt exhibited a weak gel, resulting in higher storage and loss moduli, followed by ι-carrageenan (which displayed weak gelation at 4 °C). The aforementioned observation was in line with the findings derived from the tangent loss tan δ (G″/G′) measurements, where a value exceeding 0.1 signified a characteristic feature of a weak gel [32]. However, the presence of 0.2% λ-carrageenan with 2.5% sea salt did not lead to gelation properties, resulting in both low storage modulus and loss modulus. After 24 h of fermentation, a significant decrease (*p* < 0.01) was observed in the G′ and G″ values of κ-carrageenan and carrageenan (C107615). Moreover, the tan δ of κ-carrageenan and carrageenan (C107615) at 10, 100 rad/s were changed from 0.15, 0.15 and 0.15, 0.11 to 0.12, 0.24 and 0.12, 0.11, respectively. The results indicated that the primary change observed after fermentation by *Shewanella sp.* LE8 was a reduction in the gel-forming capacity of κ-carrageenan and carrageenan (C107615) rather than an alteration in the viscoelastic properties of the gel. The G′ of ι-carrageenan also exhibited a significant decrease at lower frequencies (*p* < 0.05). Moreover, the tan δ of ι-carrageenan at 10 rad/s was increased from 0.23 to 1.19, which led to a viscous-dominant response [27]. The gel formation ability of ι-carrageenan was significantly diminished after fermentation by *Shewanella sp.* LE8, as evidenced by the combined results of apparent viscosity. This observation aligns with the phenomenon of weak gel disappearance observed in ι-carrageenan at 4 °C. The differences in G′ and G″ of λ-carrageenan before and after fermentation were negligible compared to those observed in other groups. These results also indicate that after the fermentation of carrageenan by *Shewanella sp.* LE8, the glucoside bond is broken, so that the ability of gelling is weakened or disappeared. The results also suggested that following the fermentation of carrageenans by *Shewanella sp.* LE8, the glucoside bond, undergoes cleavage, thereby leading to a reduction or loss in its gelling capacity.

### 2.3. Spectral Changes with Methylene Blue after LE8 Fermentation

**Figure 3:**
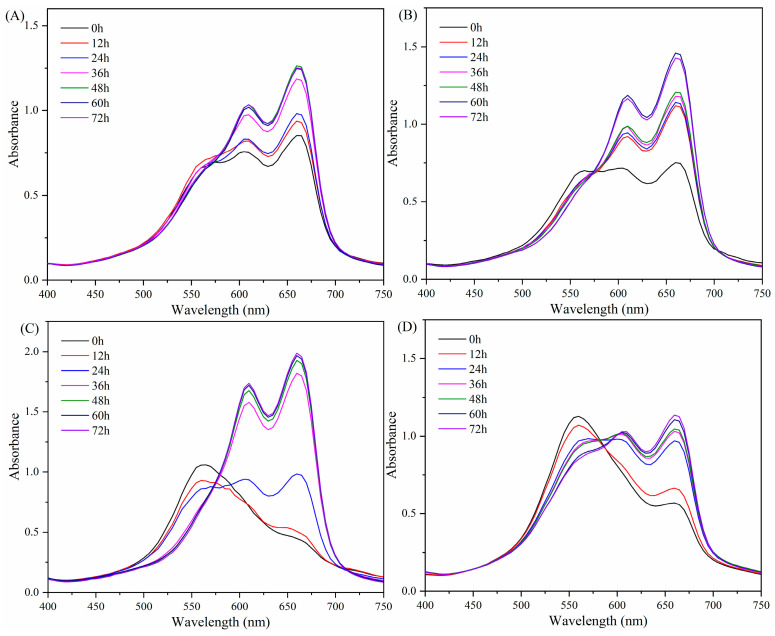
Methylene blue wavelength scanning curve of fermentation medium, with a wavelength interval of 5 nm: (**A**) carrageenan (C107615), (**B**) κ-carrageenan, (**C**), ι-carrageenan (**D**) λ-carrageenan.

Methylene Blue (MB) is a highly suitable metachromatic dye for binding with carrageenan macromolecules, and it is commonly employed in the field of food science to assess carrageenan–protein interaction using spectrophotometric techniques [33,34,35,36]. The carrageenan culture media with MB dye resulted in spectral plots presented in Figure 2. The absorption maxima of the free dye in Figure S1 were observed at 610 and 660 nm, which exhibited a high agreement with the values reported in the literature [37,38,39]. The slight variation in wave crests primarily resulted from the disparity in the minimum sweep wavelength interval. After the addition of carrageenan culture media, a newly observed absorption near 560 nm indicated the formation of a soluble metachromatic complex, which was consistent with the literature report [36]. The variation among the samples, particularly κ-carrageenan and carrageenan (C107615) to others, primarily stemmed from the disparate levels of sulfation at equivalent mass concentrations. After fermentation for 12–72 h, the new absorption near 560 nm exhibited a gradual decrease or disappearance, while absorptions at 610 and 660 nm showed a progressive increase to constant. Specifically, the most pronounced changes in ι-carrageenan were observed as a decrease in absorptions at 610 and 660 nm, along with an increase in absorption at 565 nm during the initial stage. The significant change trend can be observed within 12 to 36 h of fermentation, followed by a period of stability lasting from 48 to 72 h. It indicated that ι-carrageenan was degraded by *Shewanella sp.* LE8 during the fermentation process, which was consistent with the previous results of apparent viscosity. It is worth noting that the fermentation process of ι-carrageenan does not necessarily conclude after 48 h, as the intensity of MB dye staining relies on the quantity of carrageenan introduced. Furthermore, κ-carrageenan and carrageenan (C107615) exhibited similar fermentation characteristics, with κ-carrageenan being more readily degraded than carrageenan (C107615) within the initial 24-h fermentation period. This observation aligns with the rheological findings of previous studies regarding apparent viscosity. However, despite a gradual decrease in the absorption at 560 nm throughout the λ-carrageenan fermentation process, it remained evident even after 72 h of fermentation, and absorptions at 610 and 660 nm did not reach comparable levels as observed in the blank group (Figure 3D). It suggested that the fermentation process of λ-carrageenan might be relatively slow, and its degraded products could still form a soluble metachromatic complex with MB might be another reason. In a word, the methylene blue analysis demonstrated the degradation impact of *Shewanella sp.* LE8 on various carrageenans, with the extent of degradation being influenced by the structural characteristics of carrageenan.

### 2.4. Molecular Weights of Carrageenans after LE8 Fermentation

**Figure 4:**
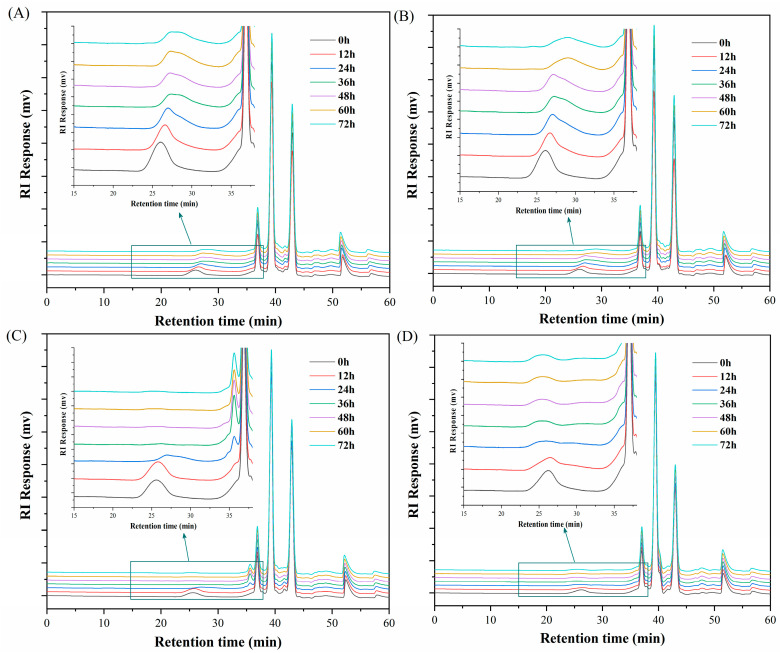
The molecular weight distribution of fermentation medium: (**A**) carrageenan (C107615), (**B**) κ-carrageenan, (**C**) ι-carrageenan, (**D**) λ-carrageenan.

The molecular weight distribution of carrageenan culture media solution during fermentation progress by *Shewanella sp.* LE8 at different time points are shown in Figure 4. The initial molecular weight of carrageenan (C107615) in the culture medium was approximately 510 kDa (Figure 4A). Following fermentation by *Shewanella sp.* LE8 for 12–36 h, a reduction in molecular weight of remaining carrageenan (C107615) to 387, 319, and 259 kDa were observed, respectively. Furthermore, no significant alteration in the molecular weight of remaining carrageenan (C107615) was detected during the fermentation period of 48–72 h. A comparable pattern was noted in the culture medium of κ-carrageenan during the initial 48-h period of fermentation. The distinction lies in the fact that during the fermentation process lasting 60–72 h, there was a further reduction in the molecular weight of remaining κ-carrageenan. At a retention time of 36 min, both carrageenan (C107615) and κ-carrageenan exhibited a negligible peak, suggesting the possible presence of degradation products by *Shewanella sp.* LE8. The products with a lower molecular weight were obscured by the peak of the culture medium, presenting another potential scenario. However, it was evident that a large number of unaltered macromolecules persist in carrageenan (C107615) and κ-carrageenan throughout the fermentation process. This finding suggested that the degradation capacity of *Shewanella sp.* LE8 towards κ-carrageenan was limited under the conditions of fermentation culture.

A totally different situation was observed in ι-carrageenan fermentation. The molecular weight distribution of ι-carrageenan did not exhibit a significant decrease (from 634 to 574 kDa) during the initial 12 h fermentation period. Subsequently, within the following 24 h, there was a rapid decline in the molecular weight of ι-carrageenan, which eventually reached a stable state after being fermented for 36 h. At this point, most of the ι-carrageenan had undergone degradation. This was consistent with the results of methylene blue analysis. Additionally, an evident and consistent peak emerged near a retention time of 35.6 min, coinciding with the disappearance of the ι-carrageenan peak (retention time of 25.6 min). The results indicate that *Shewanella sp.* LE8 was capable of fermenting ι-carrageenan to produce low molecular weight ι-carrageenan with a specific polymerization degree in the fermentation system. However, the molecular weight variation of λ-carrageenan in the fermentation process was different from other carrageenans. After a 12 h fermentation, the peak shape of λ-carrageenan underwent significant alterations, while the retention time of the main peak remained relatively unchanged. Specifically, it gradually transformed into a broad and shallow peak within the retention time range of 28–37 min. The fermentation process remained essentially unchanged for a duration of 36 h, but it was also observed that a portion of λ-carrageenan underwent limited degradation.

The variation in molecular weight among different carrageenan samples before and after fermentation suggested that *Shewanella sp.* LE8 might employ distinct degradation strategies for different types of carrageenan. Previous studies have reported the production of κ-carrageenase by *Shewanella* [20,21,22]; however, the unexpected findings from *Shewanella sp.* LE8 in relation to κ-carrageenan within the fermentation system is noteworthy. The presence of weak gel κ-carrageenan in the fermentation system may have impeded the interaction between *Shewanella sp.* LE8. Furthermore, several studies have reported that higher concentrations of Na⁺ will diminish carrageenase activity [13,16,40].

### 2.5. Changes in Total and Reducing Sugars

**Figure 5:**
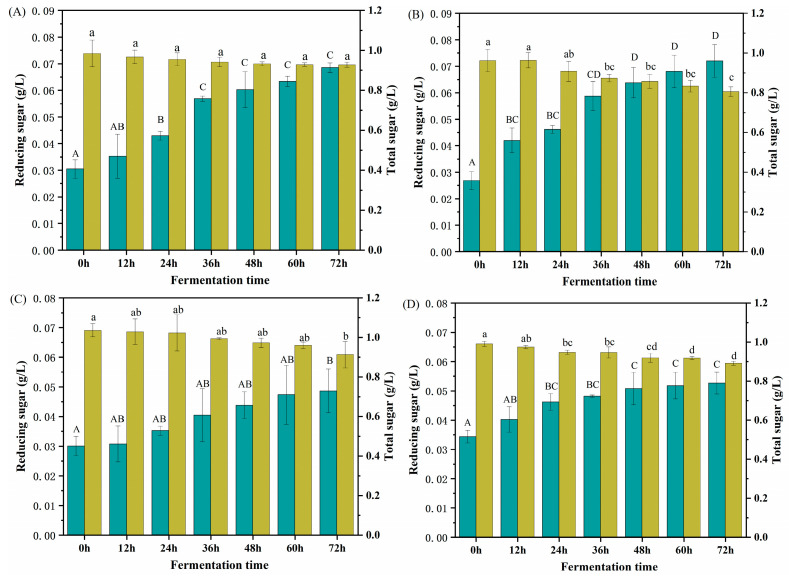
The content reducing sugar (dark cyan, left bar) and total sugar (dark yellow, right bar): (**A**) carrageenan (C107615), (**B**) κ-carrageenan, (**C**), ι-carrageenan (**D**) λ-carrageenan. Different letters (A, B, C, D) indicated significant differences in reducing sugar at different time points (*p* < 0.05); Different letters (a, b, c, d) indicated significant differences in total sugar at the different time points.

The consumption of carrageenan during fermentation and the production of reducing sugar after degradation by *Shewanella sp.* LE8 was further determined through analyses of reducing sugar and total sugar (Figure 5). The content of reducing sugar in the control group (without carrageenan addition) remains relatively stable (Figure S2), whereas all carrageenan groups exhibited an increasing trend during the entire fermentation process. The findings demonstrated that carrageenans underwent degradation through *Shewanella sp.* LE8 fermentation, resulting in the formation of polysaccharides with small molecular weight. Specifically, after 12 h of fermentation, the κ-carrageenan exhibited an increase in reducing sugar content (*p* < 0.05), which corresponded to a decrease in molecular weight. This observation further validates that the products of carrageenan (C107615) and κ-carrageenan were obscured by the peak of the culture medium (Figure 4A,B). After 72 h of fermentation, the carrageenan (C107615) and κ-carrageenan exhibited a respective increase in reducing sugar content to 2.25 and 2.69 times compared to the initial fermentation (*p* < 0.05). The reduced sugar content of carrageenan remained relatively stable during the initial 12 h of fermentation, which was consistent with the observed changes in molecular weight (Figure 4C). The reducing sugar content of ι-carrageenan and λ-carrageenan increased by 1.62 and 1.53 times (*p* < 0.05), respectively, compared to their initial levels before fermentation.

However, during the fermentation process, the control group exhibited a declining trend in total sugar content (Figure S2), which could be attributed to the utilization of carbon sources present in yeast extract powder and peptone during LE8 growth. At 72 h, the total sugar content in the medium was decreased by 0.6 g/L. The change in total sugar content of all carrageenan groups also showed a decreasing trend during fermentation, exhibited a decreasing trend during fermentation, similar to that observed in the control group. At 72 h, the total sugar content in carrageenan (C107615), κ-carrageenan, ι-carrageenan, and λ-carrageenan medium was decreased by 0.06, 0.16, 0.12, and 0.10, respectively.

The barely decreased total sugar content indicates that the utilization of carrageenan by *Shewanella sp.* LE8 during fermentation was highly limited. Therefore, employing *Shewanella sp.* LE8 fermentation for carrageenan degradation to produce oligosaccharides or polysaccharides with lower molecular weight was feasible. However, the concentration of reducing sugars was increased. Since the content of reducing sugars was one of the factors that influenced the antioxidant strength [41]. It indicated that the antioxidant activity of carrageenan would be increased after *Shewanella sp.* LE8 fermentation. In conjunction with the results from molecular weight analysis, iota carrageenan exhibited the highest degree of hydrolysis; however, its reducing sugar content was relatively low, suggesting that the fermented product of ι-carrageenan might be a small molecular weight polysaccharide rather than a monosaccharide or a small molecule sugar with high reducing properties [42]. Consequently, ι-carrageenan proves to be most suitable for generating low molecular weight polysaccharides or oligosaccharides through *Shewanella sp.* LE8 fermentation.

### 2.6. Purification and Structural Characterization of Fermentation Products

#### 2.6.1. Purification of Carrageenan Fermentation Products

**Figure 6:**
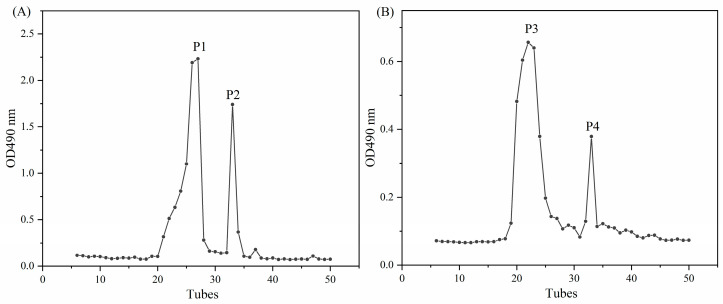
The LH-20 column chromatography elution curves of fermentation products: (**A**) ι-carrageenan, (**B**) λ-carrageenan.

The application of Sephadex LH-20 is extensive in the isolation and purification of flavonoids and oligosaccharides [43,44]. The LH-20 column chromatography elution curves of carrageenan fermentation products of ι-carrageenan and λ-carrageenan are shown in Figure 6. Both ι-carrageenan and λ-carrageenan fermentation products exhibited a prominent, broad main peak (P1 and P3) accompanied by a smaller peak (P2 and P4) representing a narrower molecular weight distribution. The hydrolyzation products of ι-carrageenases from *Alteromonas fortis*, unlike those of *Shewanella sp.* LE8, consist of a mixture comprising neo-ι-carrabiose, neo-ι-carratetraose, and neo-ι-carrahexaose [45]. However, the products generated by *Shewanella sp.* LE8 exhibits a higher degree of homogeneity, suggesting their potential suitability for various industrial applications.

Under identical fermentation and treatment conditions, the OD value of ι-carrageenan’s fermentation products was higher, indicating the generation of more low molecular weight carrageenan molecules through its fermentation process. This could be attributed to the more comprehensive fermentation of ι-carrageenan or potentially due to the partial entrapment of λ-carrageenan’s fermentation products by the 3 kDa ultrafiltration membrane envelope. These findings align well with the observed differences in molecular weight distribution during fermentation.

#### 2.6.2. UPLC-TOF MS Analysis of Carrageenan Fermentation Products

**Figure 7:**
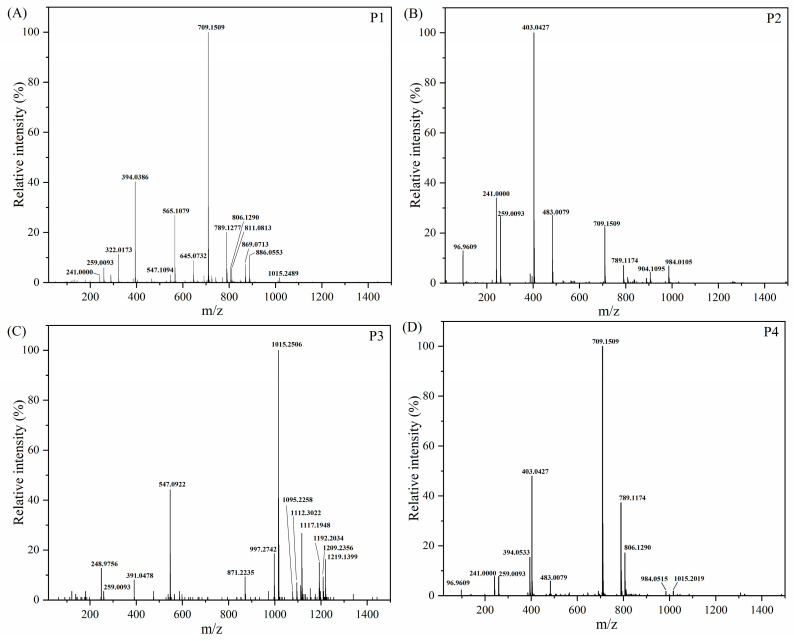
Negative ESIMS spectra of the isolated fermentation products: (**A**) P1 of ι-carrageenan, (**B**) P2 of ι-carrageenan, (**C**) P3 of λ-carrageenan, (**D**) P4 of λ-carrageenan.

ESIMS analysis of purified ι-carrageenan and λ-carrageenan fermentation products was conducted in negative mode due to the presence of sulfate anion groups, aiming to investigate the detailed structure. In the mass spectrum of P2 (Figure 7B), the ion peaks at *m*/*z* 96.96, 241.00, and 259.01 were detected, which were assigned to HSO_4_^−^, [G4S−H_2_O]^−^, G4S^−^, respectively (G4S stands for 4-sufate-β-galactose). The ion peaks at *m*/*z* 403.04, 483.01, 709.15, 789.13 were corresponding to [G4S-DA]^−^, [G4S-DA2S]^−^, [G4S-DA-G-DA]^−^, and [G4S-DA2S-G-DA]^−^ or their isomer, respectively (DA2S stands for 2-sufate-3,6-anhydro-α-d-galactose). Moreover, the ion peaks at *m*/*z* 806.13 were representative of [G4S-DA2S-G-DA+NH_4_^+^]^−^, carrying ammonium and two sulfate groups. Thus, the ion peaks at *m*/*z* 904.11 and 984.07 were probably assigned to [G4S-DA2S-G4S-DA+NH_3_·H_2_O]^−^ and [G4S-DA2S-G4S-DA2S+NH_3_·H_2_O]^−^, respectively. The results revealed that fermentation product P2 of ι-carrageenan was a tetrasaccharide composed of [G4S-DA2S]_2_.

The mass spectrum of P1 exhibited partial similarity to that of P2, albeit with the presence of additional ions, including some bivalent ions (Figure 7A). The bivalent ion peaks at *m*/*z* 322.02 and 394.04 were likely assigned to [G4S-DA-G4S]^2−^ and [G4S-DA-G4S-DA]^2−^, similar to the findings reported in the literature [46]. The new ion peaks at *m*/*z* 565.11, 645.07, and 869.07 were detected, which were assigned to [G4S-DA-G]^−^, [G4S-DA-G4S]^−^, [G4S-DA-G4S-DA2S]^−^, respectively. The ion peaks at *m*/*z* 811.08 and 886.06 were representative of [G4S-DA-G4S-DA+Na^+^]^−^, [G4S-DA-G4S-DA2S+NH_4_^+^]^−^, respectively. Furthermore, the ion peaks at *m*/*z* 1015.25 were probably corresponding to [G4S-DA2S-G4S-DA2S+3Na^+^]^−^. The MS results indicated that the fermentation product P1 of ι-carrageenan was also a tetrasaccharide composed of [G4S-DA2S]_2_.

The mass spectrum of the fermentation product P3 derived from λ-carrageenan exhibited significant differences compared to that of P1 and P2. The trivalent ion peak observed at *m*/*z* 391.05 was likely attributed to M−2H (where M−2H represented [G2S-DA]_3_^3−^ or [G-DA2S]_3_^3−^), while the bivalent ion peak at *m*/*z* 547.10 was probably corresponding to [DA-G2S-DA]_2_^2−^ or its isomer. Thus, the ion peaks at *m*/*z* 1192.20, 1209.24, and 1219.14 might be assigned to [M+NH_4_^+^]^−^, [M+2NH_4_^+^]^−^, and [M+2Na^+^]^−^, respectively. Moreover, the ion peaks at *m*/*z* 1117.19, 1112.30, 1095.23, and 1015.25 were probably representative of [M−SO_3_+Na^+^]^−^, [M−SO_3_+NH_4_^+^]^−^, [M−SO_3_]^−^, and [M−2SO_3_]^−^. The observed ion peak at *m*/*z* 997.27 was attributed to [M−2SO_3_−H_2_O]^−^, while the ion peak at *m*/*z* 871.22 was assigned to [M−DA−2SO_3_]^−^. The results indicated that the fermentation products P4 of λ-carrageenan a hexasaccharide composed of three units of [G2S-DA] or [G-DA2S]. Meanwhile, it could be inferred that the fermentation product P4 is a tetrasaccharide consisting of two units of [G2S-DA2S], as indicated by the fragment ion in Figure 7D.

It is believed that complete substrate biodegradation cannot be achieved solely by carrageenase enzymes, as it requires a diverse set of enzymes [12]. Numerous sequenced marine microbial genomes have demonstrated the abundance of sulfatase enzymes in marine bacteria, which play a crucial role in degrading algal sulfated polysaccharides like carrageenans [47]. For instance, the sulfatase isolated from the marine bacterium *Pseudoalteromonas carrageenovora* can convert ι-carrageenan into hybrid ι-/α- or pure α-carrageenan [48]. Therefore, it is possible for *Shewanella sp.* LE8 to convert λ-carrageenan into θ-carrageenan and other types of carrageenan with small molecules during the fermentation process. Despite the primary mass spectrometry results did not provide sufficient information to determine the precise structural composition. The mechanism of its sulfatase and the precise structural characterization of its products necessitate further investigation. Furthermore, the generation of θ-carrageenan oligosaccharides implies the potential for an increase in activity [49].

The utilization of carrageenan has been prevalent in food and beverages, biomedical and biotechnological industries, and pharmaceutical products as gelling agents, viscosity enhancers, and stabilizers [50]. The limited biological activity of carrageenan, as well as polysaccharides from red algae, can be attributed to these distinctive characteristics. The objective of this study was to decrease the viscosity of carrageenan using a convenient, cost-effective, and environmentally friendly approach in order to enhance its biological activity. Although the operational procedures of frequently employed acid degradation and oxidative degradation are relatively straightforward and cost-effective, the mastery of conditions and identification of degradation products throughout the entire process present challenges, thereby impacting its biological activities [51]. The enzymatic approach is characterized by high costs and inefficiency, thereby constraining its widespread adoption in industrial-scale applications [52]. Consequently, we isolated a marine microorganism to assess its capacity for degrading three typical types of carrageenans under conditions of limited nutrients. Our method offers the advantages of straightforward implementation, cost-effectiveness, and the ability to control the degradation products. The results of rheological, molecular weight, and mass spectrometry analyses substantiated that *Shewanella sp.* LE8 exhibited a discernible degradation capability towards the three varieties of carrageenan. Therefore, obtaining carrageenan with low molecular weight or oligosaccharides through *Shewanella sp.* LE8 fermentation of carrageenan and red algae polysaccharides is a viable approach. Despite concerns regarding the application of small molecule carrageenan due to associated risks of proinflammatory, it is important to note that those degradation products of κ-carrageenan reported in existing literature, known as poligeenan, primarily obtained through acid heat treatment [53]. It has been reported that acid thermal degradation of carrageenan would generate inflammation-inducing oxidative byproducts [54]. However, a growing body of literature has demonstrated that degraded carrageenan exhibits enhanced antioxidant, anti-tumor, and anti-inflammatory activities [4,7,23,55]. Given the specific structure of carrageenan oligosaccharides, it can be inferred that the carrageenan prepared in this study possesses similar structural characteristics to those reported in the existing literature. Therefore, *Shewanella sp.* LE8 exhibits promising application prospects, particularly in the depolymerization of iota and lambda carrageenan, which have received limited attention, as well as complex polysaccharides derived from red algae.

## 3. Conclusions

The effects of a novel marine bacterium, *Shewanella sp.* LE8, on rheological properties, molecular weight distribution of three representative carrageenans (κ-, ι-, and λ-carrageenan), as well as structural characteristics of fermentation products, were investigated. After fermentation for 24 h, the apparent viscosity, storage modulus, and loss modulus of carrageenans were rapidly decreased. The results of methylene blue and molecular weight distribution analyses revealed that *Shewanella sp.* LE8 exhibited the highest degradation capacity on ι-carrageenan, followed by λ- and κ-carrageenan during the fermentation period of 24–72 h. The optimal fermentation durations for the production of oligosaccharides ranged from 48 to 72 h. The two purified components obtained through a 72-h fermentation of ι-carrageenan are both tetrasaccharides, whereas the two purified components derived from the fermentation of λ-carrageenan were hexasaccharide and tetrasaccharide, respectively. Therefore, *Shewanella sp.* LE8 exhibits promising application prospects, particularly in the depolymerization of multiple carrageenans, as well as complex polysaccharides derived from red algae.

## 4. Materials and Methods

### 4.1. Materials

The carrageenan (C107615), κ-carrageenan (C121013), ι-carrageenan (C120988), and λ-carrageenan (C121014) were purchased from Aladdin Biochemical Technology Co., LTD, Shanghai, China. The *Shewanella sp.* LE8 (CCTCC M 20231549) was isolated from the intestines of abalone purchased from Wuxi, Jiangsu, China. The 2216E liquid medium (HB0132-1) was purchased from Hope Bio-Technology Co., Ltd., Qingdao, Shandong, China.

### 4.2. Culture Medium and Growth Conditions

The *Shewanella sp.* LE8 was cultured in 70% 2216E medium containing 37.4 g/L 2216E liquid medium and was incubated at 26 °C for activation. A base medium composed of 25 g/L sea salt, 2.5 g/L peptone, and 1 g/L yeast extract with pH 7.2 was used. The 0.2% (m/v) of carrageenan, κ-carrageenan, ι-carrageenan, and λ-carrageenan were added into the base medium separately. Then, all mediums were sterilized at 121 °C for 15 min before use. The 2% (*v*/*v*) of activated *Shewanella sp.* LE8 was inoculated into carrageenan mediums and incubated at 28 °C for 0–72 h. Among them, the carrageenan and κ-carrageenan mediums were inoculated in a liquid state. During the culture process, implementing a gentle oscillation every 12 h facilitates more uniform growth of LE8. After the culture points were terminated, the medium was subsequently exposed to a temperature of 99.5 °C for 20 min in order to halt the cultivation process and inactivate the enzyme.

### 4.3. Dynamic Viscosity and Rheological Analysis

The dynamic viscosity of carrageenan culture mediums (24 h of cultivation) was determined by a method described previously with some modifications [56,57]. Briefly, the carrageenan culture mediums were subjected to enzyme deactivation through heating at 99.5 °C for 20 min, followed by cooling to room temperature. Then, carrageenan culture mediums were loaded on the plate of fluids rheometer DHR-3 (TA Instruments Ltd., New Castle, DE, USA) with parallel plates of a diameter of 45 mm and gap value of 1 mm at a shearing rate of 0.1–100 s^−1^ at 25 °C. The storage modulus (G′) and loss modulus (G″) of carrageenans were determined at the same conditions, where the stress was 0.8%.

### 4.4. Methylene Blue Analysis

The methylene blue analysis was conducted with some modifications based on published methods [33,36,38]. In brief, 0.2 mg/mL sample solution (20 μL, carrageenan culture mediums with 0–72 h of cultivation) with 10 mg/mL methylene blue (180 μL) were mixed, and the wavelength of maximum absorption (λmax) of mixtures was performed over from 400 to 750 nm with a microplate reader (FilterMax F5, Molecular Devices Co, San Francisco, CA, USA).

### 4.5. Determination of Molecular Weights

The molecular weight (Mw) distributions of carrageenans (carrageenan culture mediums with 0–72 h of cultivation) were determined using high-performance gel permeation chromatography (HPGPC, Waters e2695, Waters, Massachusetts, USA) with a Waters 2414 refractive index detector [57,58]. The TSK-GEL columns (Tosoh Co., Ltd., Tokyo, Japan) were in series with TSK-GEL guard column (6.0 × 40 mm), GMPWXL (300 × 7.8 mm) and G3000PWXL (300 × 7.8 mm). The dilute carrageenans solutions were eluted with NaNO_3_ (0.1 M) at a flow rate of 0.5 mL/min after being filtered by a 0.45 μm aqueous phase filter. The injection volume was 20 μL in each run. The column and the detector temperature were maintained at 40 °C. The molecular weight of carrageenans was determined by measuring the elution volume, which is directly proportional to the logarithm of the molecular weight of standard dextrans.

### 4.6. Determination of Total and Reducing Sugars

The content of total carbohydrates was quantified using the phenol-sulfuric acid method with glucose as the reference standard [59]. The content of reducing sugars was determined at 540 nm using the dinitrosalicylic acid (DNS) method with glucose as standard [60].

### 4.7. Structural Analysis of Carrageenans Fermentation Products

#### 4.7.1. Preparation and Purification of Polysaccharides from Fermentation Products

The base medium was supplemented separately with 0.2% (*w*/*v*) of ι-carrageenan and λ-carrageenan, followed by inoculating 3% (*v*/*v*) of activated *Shewanella sp.* LE8 and fermented at 28 °C for 72 h. After the completion of fermentation, the culture media were subjected to enzyme inactivation by heating at 99.5 °C for a duration of 20 min. The samples were subsequently filtered using a 0.45 μm PVDF aqueous phase filter, and the resulting filtrates were collected. These filtrates were then separated by employing a 3 kDa ultrafiltration membrane (Vivaflow 200, Sartorius Corporation, Goettingen, Germany). Subsequently, the filtrates (<3 kDa fraction) were concentrated at 50 °C using a rotary evaporator (RV8, IKA Corporation (IKA-Werke GmbH & CO. KG), Staufen, Germany), followed by precipitation at 4 °C for 12 h. The precipitates were subsequently subjected to vacuum filtration through a 0.22 µm organic filter and washed with ethanol. Then, the precipitations were re-dissolved in ddH_2_O and were further purified by an exclusion chromatography of Sephadex LH-20 with ddH_2_O as the eluent at a flow rate of 0.5 mL/min. The eluent was collected in each tube with a volume of 2 mL for the purpose of total sugar content detection.

#### 4.7.2. UPLC-TOF MS Analysis

The purified fermentation products were subjected to UHPLC-TOF/MS analysis. The chromatographic separation was performed on a BEH C18 column (1.7 um, 2.1 × 150 mm) with the following eluent gradient: 100% formic acid (0.1%) for 40 min, followed by a linear gradient of 30% acetonitrile and 70% formic acid (0.1%) over the next 45 min, then an increase to 80% acetonitrile and 20% formic acid (0.1%) in the subsequent 50 min, and finally reaching 100% acetonitrile at 55 min before returning to 100% formic acid (0.1%) as the final step. The flow rate was set at 0.3 mL/min, and the column temperature was maintained at 45 °C. Mass spectrometry conditions included a gas temperature of 400 °C, a flow rate of 12 L/min, a capillary voltage of 3500 V, and a nozzle voltage of 1800 V. The mass range analyzed ranged from 20 to 1500 *m*/*z*.

### 4.8. Statistical Analysis

The data were expressed as the mean ± standard deviation (SD) of repeated analyses (n = 3). Statistical analysis used a one-way ANOVA procedure, and then Tukey and Duncan multiple comparison tests were performed using SPSS 20 software (IBM Corp., Armonk, NY, USA). A significance level of *p* < 0.05 was considered statistically significant, while a significance level of *p* < 0.01 was considered highly significant.

## Data Availability

The original contributions presented in the study are included in the article/supplementary material; further inquiries can be directed to the corresponding author/s.

## Supplementary Materials

The following supporting information can be downloaded at https://www.mdpi.com/article/10.3390/gels10080502/s1, Figure S1: Methylene blue wavelength scanning curve of fermentation medium without carrageenan; Figure S2: The content reducing sugar and total sugar of fermentation medium without carrageenan; Figure S3: The amplified view of some ion fragments.
