# Supplementary material for: Rheological and Structural Characterization of Carrageenans during Depolymerization Conducted by a Marine Bacterium Shewanella sp. LE8
Source: Gels. 2024 Jul 28;10(8):502. doi: 10.3390/gels10080502 (PMC11353762; doi:10.3390/gels10080502)
Supplement: Supplementary file 1 [file gels-10-00502-s001.zip › gels-3116444-supplementary.pdf]

Supplementary materials

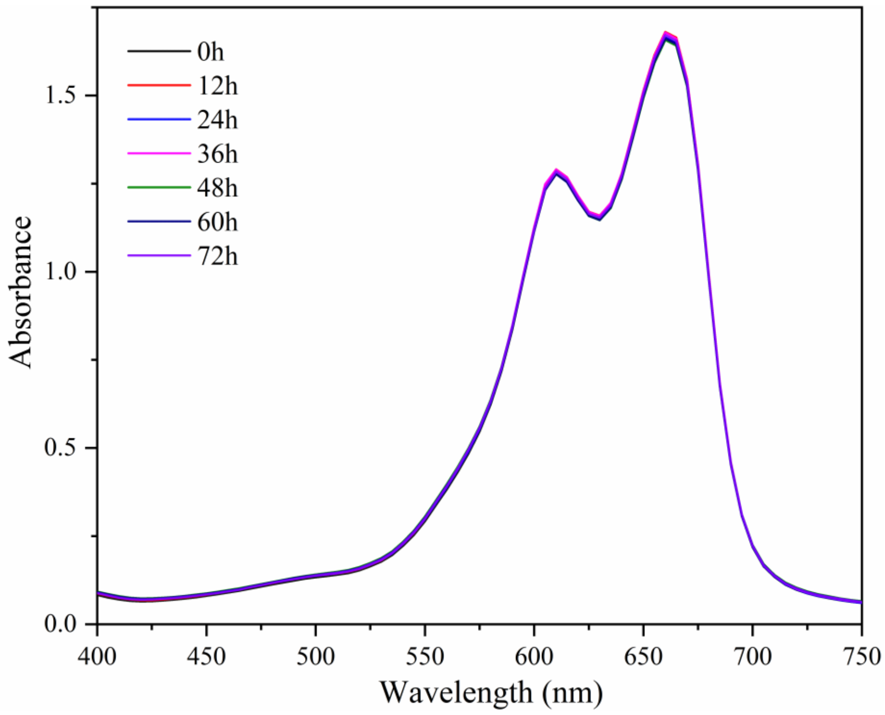

Fig. S1. Methylene blue wavelength scanning curve of fermentation medium without carrageenan.

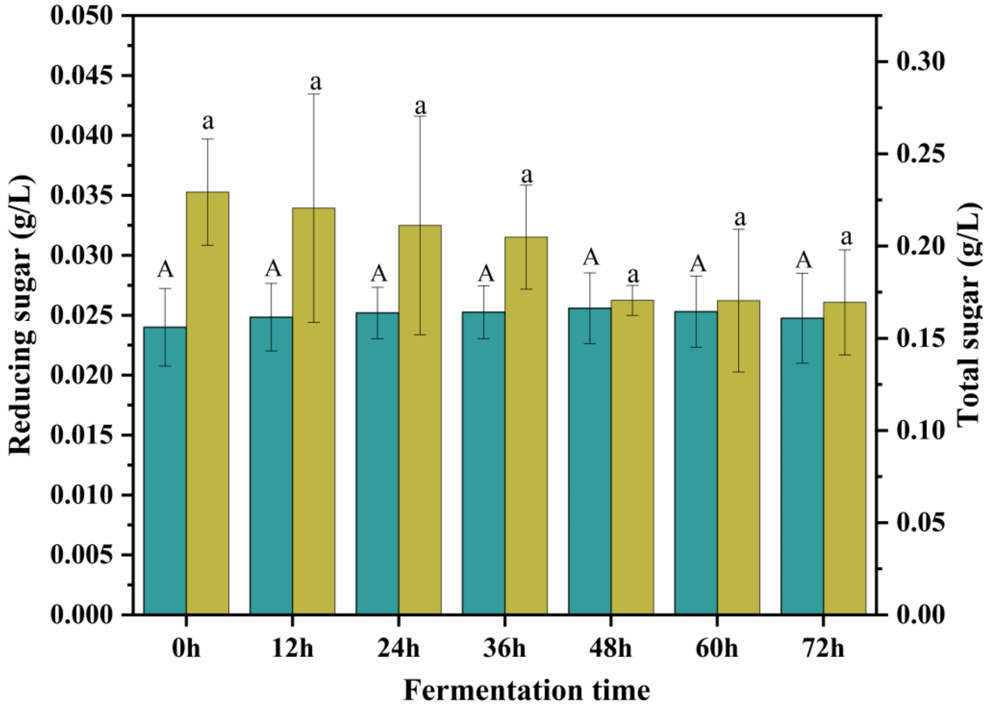

Fig. S2. The content reducing sugar and total sugar of fermentation medium without carrageenan.

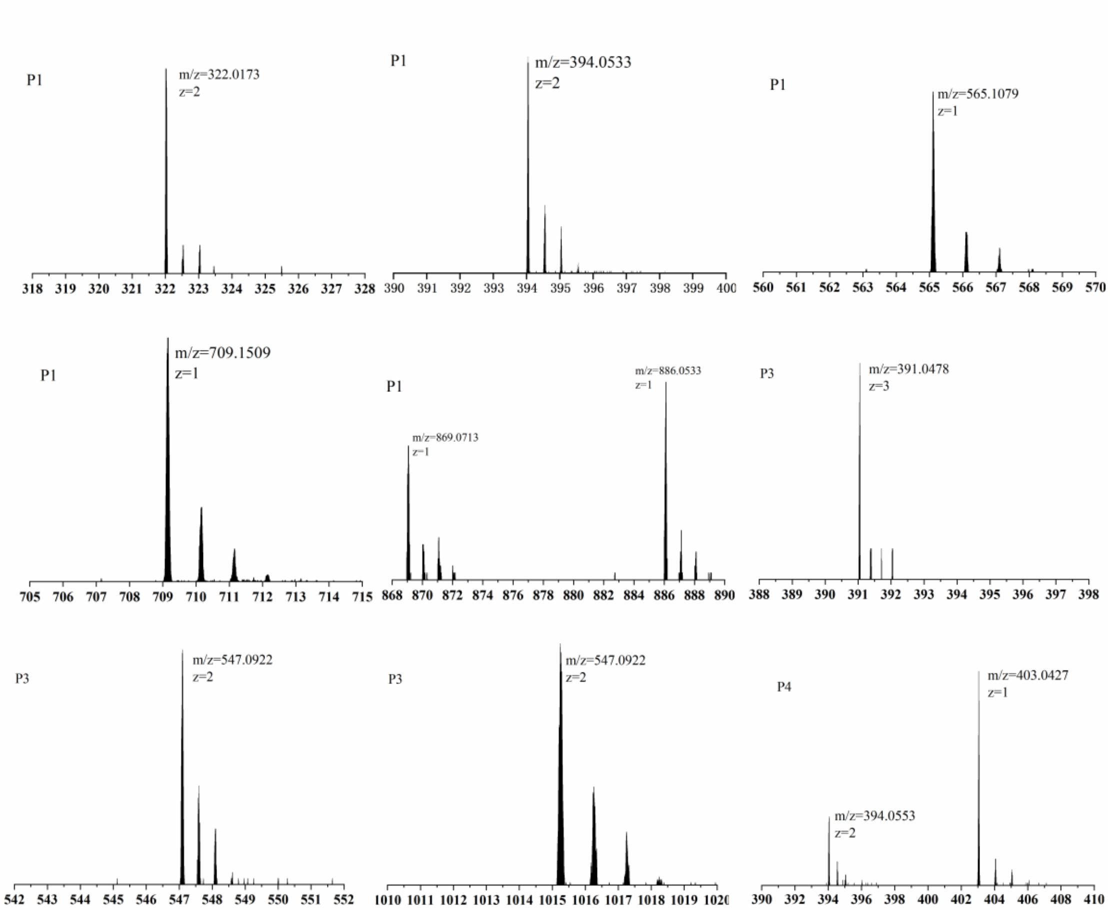

Fig. S3. The amplified view of some ion fragments.
